# Pyometra, an Unusual Case of Acute Abdomen

**DOI:** 10.5811/cpcem.2018.5.38221

**Published:** 2018-07-12

**Authors:** Kyle E. Nielsen, Sarah A. Medeck, Dan B. Brillhart, Kasey J. Mayclin

**Affiliations:** Carl R. Darnall Army Medical Center, Department of Emergency Medicine, Fort Hood, Texas

## Abstract

A 49-year-old female six days post-endometrial biopsy presented to the emergency department with constant severe suprapubic abdominal pain, fevers and myalgia. A contrasted computed tomography noted an intrauterine fluid collection and a final diagnosis of pyometra was made in the operating room following total hysterectomy. Pyometra is an exceptionally rare clinical condition with significant mortality.

## INTRODUCTION

Non-traumatic abdominal pain is one of the most common reasons for emergency department (ED) visits. It is also a notoriously difficult complaint to approach in females due to the myriad of abdominal and pelvic etiologies. In female patients, pelvic pathology accounts for approximately 12% of acute abdominal pain presentations.^1^ This case report illustrates an otherwise healthy female who, due to a unique series of events, developed an intrauterine infection that progressed to a rare surgical emergency called a pyometra.

## CASE REPORT

A 49-year-old female presented to the ED with diffuse abdominal pain, fevers, myalgia and nausea. The patient had an unsuccessful cervical dilation and endometrial biopsy six days prior to presentation. She was seen in gynecology clinic on post-procedure day two and was started on oral metronidazole for suspected bacterial vaginosis due to a foul-smelling discharge, which subsequently resolved. Pertinent surgical history included an endometrial ablation and bilateral tubal ligation.

**Image 1:**
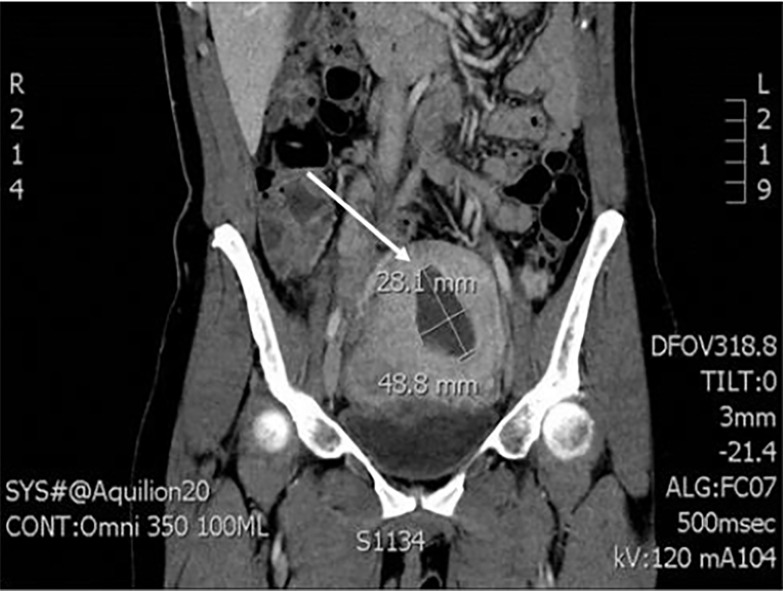
Contrasted computed tomography with the white arrow demonstrating the 2.8 cm × 4.8 cm intrauterine fluid collection.

**Image 2:**
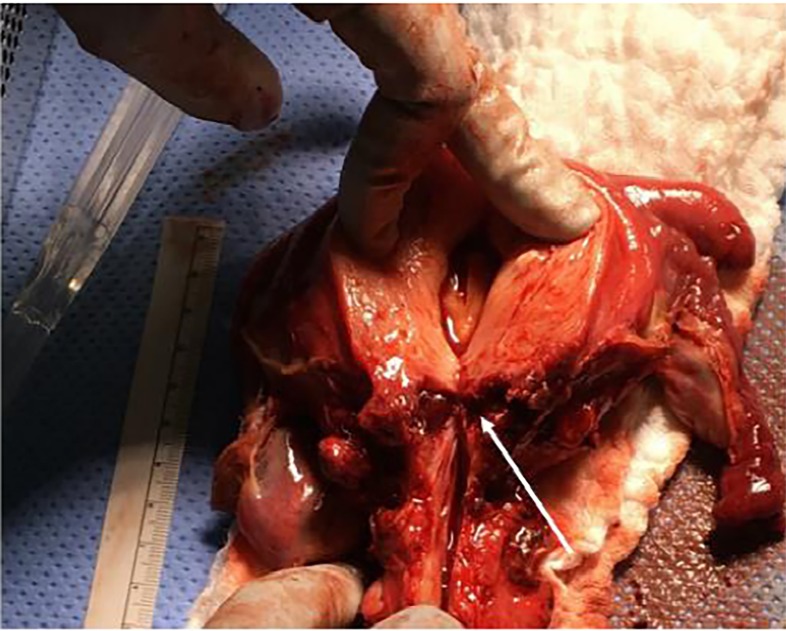
Tense fluid in the uterine cavity ruptured when bi-valved. White arrow illustrates the cervical canal is completely obliterated due to patient’s prior ablation.

On arrival, the patient was mildly tachycardic but otherwise hemodynamically stable and afebrile. She was ill-appearing. On physical exam, severe diffuse abdominal tenderness and guarding was noted. A pelvic exam noted uterine tenderness and scant dark blood in the vaginal vault, but without appreciable discharge. Laboratory results were significant for mild leukocytosis with white blood cell count of 12 × 10^3/μL (ref 3.98–10.04) but otherwise unremarkable. Her contrasted abdominal and pelvis computed tomography demonstrated a 2.8 cm × 4.8 cm intrauterine fluid collection (Image 1). Ampicillin, clindamycin and gentamycin were started. Gynecology was consulted and patient was taken to the operating room for emergent dilation and curettage. The procedure was unsuccessful due to complete cervical stenosis and severe uterine tissue inflammation and edema. Repeated ultrasound-guided attempts failed, and a non-perforating iatrogenic false lumen was created in the posterior myometrium. The following day, the patient was taken back to the operating room for a total abdominal hysterectomy. The surgeon reported a tense, fluid-filled uterus that ruptured when bi-valved, consistent with a pyometra (Image 2).

## DISCUSSION

Pyometra is an intrauterine infection and collection of purulent material due to the inability of the cervix to adequately drain the uterine contents. It is a well-known entity within the veterinary community due to its relative frequency in dogs and cattle,^2^ but it is exceptionally rare in humans. A 2014 literature review found only 81 reported cases from 1949–2015, and noted a mortality rate of 31%.^3^ *Streptococcus* species, *Bacteroides fragilis* and *Escherichia coli* are the most common organisms isolated in pyometra.^4^,^5^ In the few reported human cases, the majority were caused by cervical obstruction due to malignancy.^2^

Our patient developed a hematometra (retention of blood in the uterus) due to her prior uterine ablation and resultant cervical stenosis.^1^ Her prior bilateral tubal ligation, which prevented retrograde expulsion of the menses into the abdominal compartment, likely also contributed to her presentation. The chronic collection of stagnant proteinaceous bodily fluid likely served as an excellent medium for bacteria introduced via instrumentation during the patient’s attempted endometrial biopsy six days prior. Although this patient’s intrauterine cultures grew negative, by the time they were obtained the patient had been on ampicillin, gentamicin, and clindamycin for over 24 hours. The patient’s symptoms resolved and she was discharged on hospital day three with gynecology follow-up.

**CPC-EM Capsule**

What do we already know about this clinical entity?

Pyometra is a serious, life-threatening infection within the uterus. It is typically caused by anaerobic bacteria growth within a poorly draining uterus.

What makes this presentation of disease reportable?

Pyometra, although a well-known entity within the veterinary community due to frequency in dogs and cattle, is exceptionally rare in humans.

What is the major learning point?

Early broad spectrum antibiotics to include anaerobic coverage and urgent surgical intervention are important in the treatment of pyometra.

How might this improve emergency medicine practice?

Increased awareness of rare abdominal pathology will allow for future recognition and management.

## CONCLUSION

This case was resultant from the culmination of a unique series of events including prior tubal ligation, cervical stenosis with development of a chronic hematometra and ultimately iatrogenic inoculation via recent instrumentation. As emergency physicians, we are often asked to make decisions on incomplete data. Although the ultimate diagnosis of pyometra was made in the operating room over 24 hours later, this patient benefited from early, broad-spectrum antibiotics and fluid resuscitation. Furthermore, this case illustrates the need to maintain a broad differential when evaluating nontraumatic abdominal pain. In summary, we report an exceptionally rare case of a pyometra in a human. To our knowledge, this has not been reported within the emergency medicine literature.

Documented patient informed consent and/or Institutional Review Board approval has been obtained and filed for publication of this case report.
